# Physics-Based Graphics Models in 3D Synthetic Environments as Autonomous Vision-Based Inspection Testbeds

**DOI:** 10.3390/s22020532

**Published:** 2022-01-11

**Authors:** Vedhus Hoskere, Yasutaka Narazaki, Billie F. Spencer

**Affiliations:** 1 Department of Civil and Environmental Engineering, University of Houston, Houston, TX 77024, USA; 2 Department of Civil and Environmental Engineering, ZJUI-UIUC Institute, Zhejiang University, Hangzhou 310027, China; narazaki@intl.zju.edu.cn; 3 Department of Civil and Environmental Engineering, University of Illinois at Urbana-Champaign, Urbana, IL 61801, USA; bfs@illinois.edu

**Keywords:** inspection testbeds, deep learning, computer graphics, autonomous inspections, physics-based graphics models, damage detection

## Abstract

Manual visual inspection of civil infrastructure is high-risk, subjective, and time-consuming. The success of deep learning and the proliferation of low-cost consumer robots has spurred rapid growth in research and application of autonomous inspections. The major components of autonomous inspection include data acquisition, data processing, and decision making, which are usually studied independently. However, for robust real-world applicability, these three aspects of the overall process need to be addressed concurrently with end-to-end testing, incorporating scenarios such as variations in structure type, color, damage level, camera distance, view angle, lighting, etc. Developing real-world datasets that span all these scenarios is nearly impossible. In this paper, we propose a framework to create a virtual visual inspection testbed using 3D synthetic environments that can enable end-to-end testing of autonomous inspection strategies. To populate the 3D synthetic environment with virtual damaged buildings, we propose the use of a non-linear finite element model to inform the realistic and automated visual rendering of different damage types, the damage state, and the material textures of what are termed herein physics-based graphics models (PBGMs). To demonstrate the benefits of the autonomous inspection testbed, three experiments are conducted with models of earthquake damaged reinforced concrete buildings. First, we implement the proposed framework to generate a new large-scale annotated benchmark dataset for post-earthquake inspections of buildings termed QuakeCity. Second, we demonstrate the improved performance of deep learning models trained using the QuakeCity dataset for inference on real data. Finally, a comparison of deep learning-based damage state estimation for different data acquisition strategies is carried out. The results demonstrate the use of PBGMs as an effective testbed for the development and validation of strategies for autonomous vision-based inspections of civil infrastructure.

## 1. Introduction

**Figure 1:**
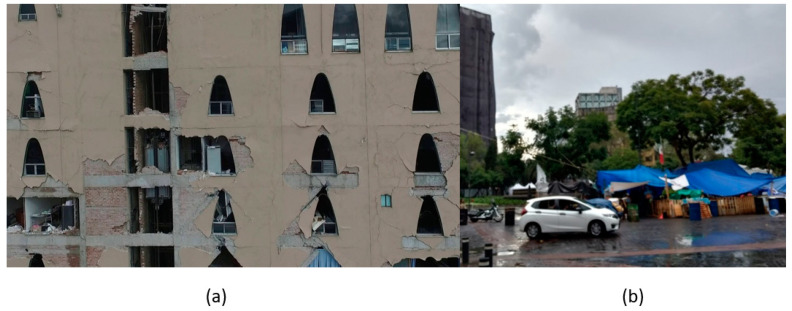
(**a**) Building damaged during the 2017 Puebla Earthquake. (**b**) Temporary tents by citizens evacuated from buildings under inspection.

The inspections of structures that are necessary after earthquakes are laborious, high-risk, and subject to human error. Describing the nature of inspections in a post-disaster scenario, the ATC-20 field manual [1] states that post-earthquake safety evaluations of buildings are “grueling work,” resulting in a high level of stress on the volunteer inspectors that may lead to “burn-out.” Entry into damaged structures for inspections poses a serious safety risk due to the unknown nature of their structural integrity. Additionally, the time-consuming nature of these inspections can exacerbate the social and economic impacts of the disaster on affected communities. For example, immediately after the 2017 Central Mexico Earthquake, hundreds of thousands of citizens had to exit damaged buildings (Figure 1a) and were left with uncertainty about the state of their homes and offices [2]. The initial evaluations conducted by the Civil Engineering Association took three weeks from the occurrence of the earthquake [3]; at that time, 1210 buildings showed signs of damage (e.g., Figure 1a), but were safe to occupy, 327 buildings were severely damaged and unsafe to occupy, and 427 buildings needed detailed evaluation [4]. Nearly 17,000 people were being housed in camps for weeks [5] while waiting for detailed inspections [6], and hundreds could still be seen still camping out in tents after six months (Figure 1b). Similar scenarios may be observed anytime a large earthquake hits a densely populated region [7,8,9]. To help communities recover swiftly after a disaster and get people back in their homes and businesses as soon as possible, more efficient inspections are required. 

Data acquisition with unmanned aerial vehicles (UAVs) and data processing using deep learning algorithms have shown tremendous potential in advancing the level of autonomy in post-earthquake inspections. A frequently studied problem is the application of machine learning algorithms, with a specific focus on the use of deep convolutional neural networks (CNNs) for damage identification after earthquakes. Yeum et al. [10] proposed the use of region-based R-CNN for spalling detection in earthquake-damaged buildings. Mondal et al. [11], implemented the Faster R-CNN [12] algorithm to compare different network architectures for multi-class detection of damage in earthquake-affected buildings. Xu et al. [13] utilized Faster R-CNN for damage identification of cracks, spalling, and exposed rebar in concrete columns. Researchers have also sought to incorporate the context of the damage and information from the entire structure to contribute to a structural assessment using deep learning methods. For example, Hoskere, et al. [14,15] proposed the use of deep-learning based semantic segmentation for multiple types of damage and materials. The proposed methodology was extended to the semantic segmentation of scenes, components, and damage in reinforced concrete (RC) buildings in [16]. Narazaki et al. [17,18] proposed the use of fully convolutional neural networks to identify bridge components for post-earthquake inspections. Narazaki et al. [19] employed recurrent neural networks with video data to help better understand the structural component context of close up videos during bridge inspections. Gao et al. [20] developed the PEER-Hub dataset incorporating multiple classification challenges for the post-earthquake assessment of buildings. Liang et al. [21] proposed a three–level image–based approach for post–disaster inspection reinforced concrete bridges using deep learning. Dizaji et al. [22] conducted preliminary research on using 3D data to train a network for defect identification of cracks and spalling on concrete columns. Pan et al. [23] presented a framework to combine performance assessment with repair cost evaluation using deep learning, extending the types of information that can be extracted from image data to aid decision-makers. A detailed review of advances in vision-based inspection and monitoring can be found in Spencer et al. [24]. 

The significant progress in computer graphics software over the past decade has allowed the creation of photo-realistic images and videos from 3D synthetic environments that have spurred advances in computer vision. The data generated from these graphics models, termed synthetic data, provides the ability to rapidly generate a large amount of diverse data (e.g., representing different geometries, colors, lighting, viewpoints, etc.) that may be impossible to otherwise acquire in the real world. Synthetic data has been used to validate applications like robotic simulation (e.g., Gazebo [25]) and for reinforcement learning in autonomous vehicles (e.g., AirSim [26]). Such synthetic data has also been used for semantic segmentation of urban scenes that have shown promising performance on field-collected data (Ros et al. [27]). Moreover, improving diversity and photorealism of the simulated worlds has helped improve the performance of methods trained on synthetic data and subsequently applied on field data, as demonstrated by recent results in self-driving [28,29,30]. 

Researchers have recently begun utilizing 3D synthetic environments for applications in vision-based inspection and monitoring. Hoskere et al. [31,32,33] proposed ideas on physics- and heuristics-based damage models as inspection testbeds and demonstrated them for inland navigation infrastructure. For structural monitoring applications, Narazaki et al. [34,35] also developed physics-based graphics models for displacement and strain measurement of inland navigation infrastructure and laboratory bridge structures. Zdziebko et al. [36] developed a physics-based graphics model of a laboratory beam structure for the development of vision-based displacement measurement algorithms. For post-earthquake inspections of RC viaducts, Narazki et al. [37] proposed heuristics-based models in a 3D synthetic environment to obtain a dataset of images and train a deep neural network for damage detection. While the efficacy of deep learning methods has been demonstrated for autonomous inspection subtasks (such as data acquisition, damage identification, and decision making), for robust real-world applicability, these subtasks need to be addressed in an integrated manner, incorporating diverse scenarios including variations in structure properties (e.g., geometry, color, material properties, damage amount), loading (intensity, frequency content, etc.), camera properties (distance, viewpoint, etc.), and environment (lighting, surrounding objects). 

**Figure 2:**
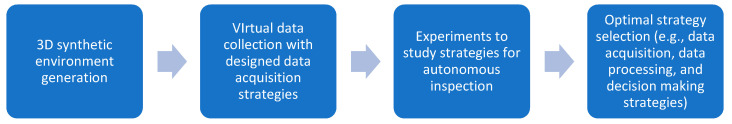
Flowchart of the proposed testbed process: After the generation of 3D synthetic environments, virtual data is collected and can be used to study and select different autonomous inspection strategies.

This paper proposes a novel framework for automatically generating 3D synthetic environments spanning diverse scenarios in structure properties, loading, camera properties, and environment necessary for a robust inspection testbed. In particular, we propose a procedure to generate physics-based graphics models (PBGMs) that incorporate a finite element model with non-linear time history analysis for modeling the response of a structure, and novel graphics algorithms to render physically consistent damage. Another significant contribution of our research is the demonstration of the utility of the proposed testbed through experiments with reinforced concrete RC buildings subject to earthquake excitation. First, we implement the proposed framework to generate a new large-scale annotated benchmark dataset for post-earthquake inspections of buildings termed QuakeCity. Second, we demonstrate the improved performance of deep learning models trained using the QuakeCity dataset for inference on real data. Finally, a comparison of deep learning-based damage state estimation for different data acquisition strategies is carried out. A general flowchart of the testbed process is provided in Figure 2. The manuscript is organized into the following sections, (i) 3D synthetic environments for inspections, (ii) implementation of the proposed framework for RC buildings, (iii) applications and experiments, (iv) results, and (v) conclusions followed by references. 

## 2. Physics-Based Graphics Models in 3D Synthetic Environments 

**Figure 3:**
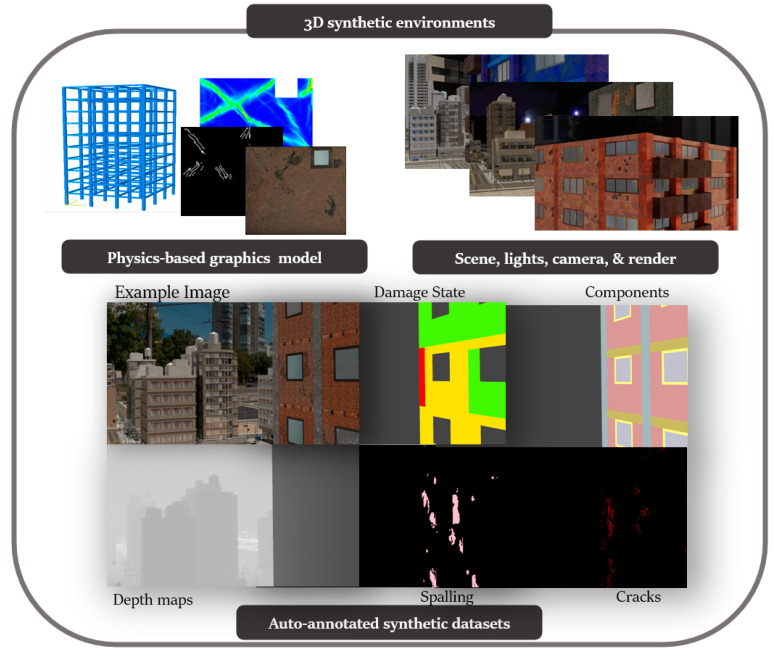
3D synthetic environments.

**Figure 4:**
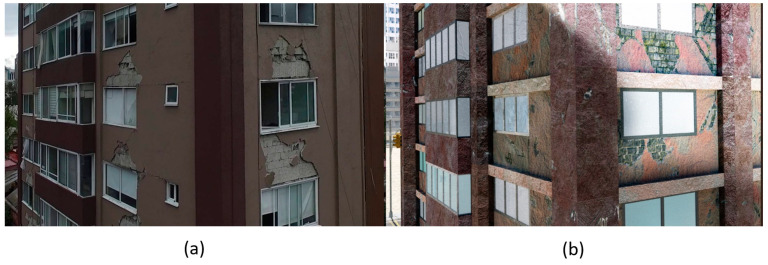
(**a**) Real image of a building damaged after the Puebla Earthquake captured from a UAV (**b**) synthetic PBGM image of a building with similar layout subject to the Tabas earthquake with different lighting generated using the proposed approach.

**Figure 5:**
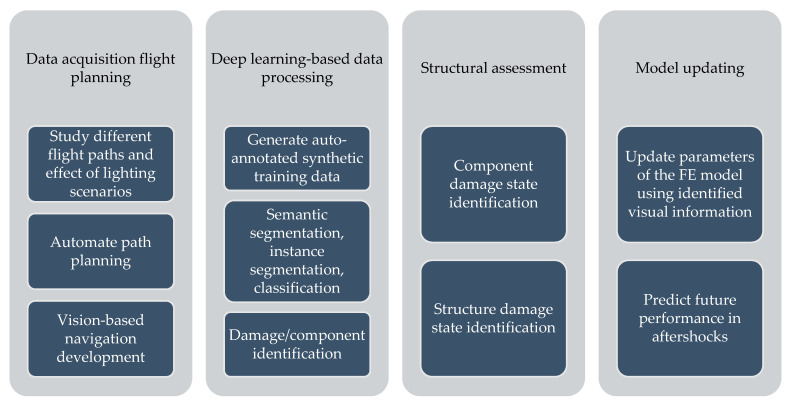
Applications of PBGMs in synthetic environments for autonomous vision-based structural inspections.

3D synthetic environments (Figure 3) are defined as modeling software with the ability to simulate object geometries and textures, lighting sources, and cameras. Using synthetic environments, image capture from UAV during an inspection is simulated by rendering images from camera locations following planned flight trajectories. Different flight paths and data acquisition schemes can be evaluated in the synthetic environment for identification of flight parameters like distance from the structure for optimal identification accuracy of both damage and components, flight path for complete coverage, etc. Before such tests can be carried out, a key challenge is to model the structure and environment of interest. In this study, PBGMs are proposed as an effective tool for modeling the structures of interest in 3D synthetic environments. Generation of synthetic data using PBGMs allows for the creation of useful annotated datasets of damaged structures, as any data from algorithmically generated graphics models will be automatically labeled, both at the pixel and image-level using damage locations and states implicit in the PBGM. Different conditions, such as ground excitation, lighting, paint colors, dirt, etc. can be simulated, to generate a wider variety of training data robustly representing different realistic environments (Figure 4). The generated data can be used to train a deep network for semantic segmentation, facilitating the automation of multiple tasks. As the damage is informed by a finite-element model, the generated data can be used to conduct overall assessments using the ground truth of the structure condition is available. Finally, as the visual representations are linked to the results of the finite element model, they provide one means of developing finite element model updating strategies. Figure 5 lists applications of PBGMs in synthetic environments for various visual inspection tasks. PBGMs and synthetic environments will provide a testbed for vision-algorithms with readily repeatable conditions. Algorithms that are effective in these virtual testbeds will be more likely to work well on field-collected datasets. The developed datasets using can also be used to augment field datasets to enhance accuracy. 

**Figure 6:**
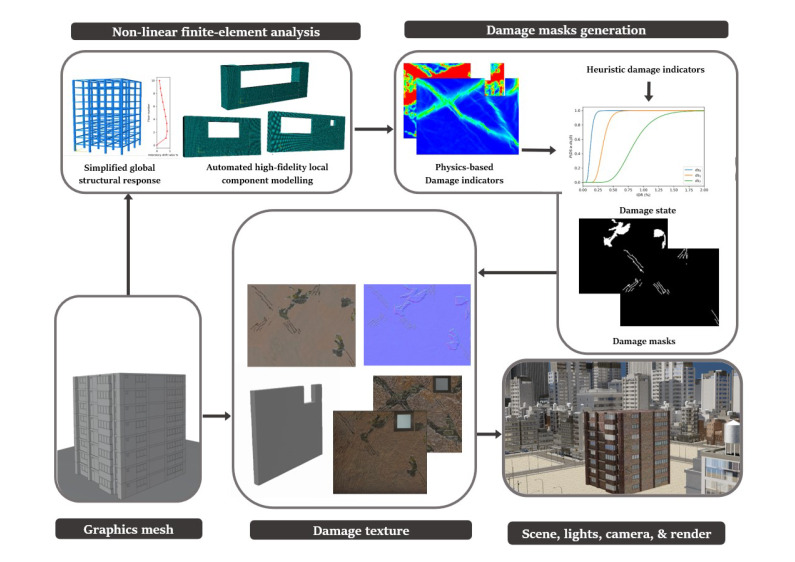
Framework for generation of PBGMs in 3D synthetic environments.

A framework for the generation of physics-based graphics models (PBGMs) for inspections is now presented. For clarity, the framework is illustrated in a schematic presented in Figure 6 with reinforced concrete buildings with masonry infill walls as the structure type. However, the same procedures may be followed for other structures where the physics can be simulated through finite element models. The framework consists of five steps including, (i) graphics mesh, (ii) non-linear finite element analysis, (iii) damage masks generation (iv) damage texture, and (v) scene, lights, camera, and render.

### 2.1. Graphics Mesh 

The geometry of the structure of interest in the PBGM is represented by a 3D mesh. The mesh may be created in any 3D creator software. The features of buildings incorporated in the 3D mesh will enable networks trained on synthetic data to learn representations for those features in real images. For structural inspections of buildings, structural components like beams, columns, and shear walls, and non-structural components like infill walls, doors, windows, and balconies, are highly relevant as damage to these components provides visual indicators of structural health. Similar lists can be made for other types of structures to be inspected. All these components should be created programmatically through parameterization, or, as referred to in the field of computer graphics, created “procedurally”. Procedural generation of the mesh will allow programmatic implementation of subsequent steps, thus enabling randomization of both geometry and textures. Randomization has been shown to improve the performance for related tasks like robotic perception when learning from synthetic data by Tobin et al. [38] and is regarded as an effective way to learn generalizable representations [29]. 

### 2.2. Non-Linear Finite Element Analysis

From the perspective of PBGM generation, non-linear finite element analyses provide valuable insight into the regions in a structure where damage is most likely to occur. The same parameters used to construct the mesh procedurally are used to generate finite element models as well. In the particular case of post-earthquake inspections, a two-step analysis approach is proposed, first obtaining a simplified global response of the structure and then conducting a high-fidelity analysis for the visible components to generate accurate damage patterns. The main pieces of information derived from these analyses are the plastic strain contours, and other damage indicators such as the compression damage index from a concrete damaged plasticity model, which provides direct indicators for cracking and spalling of members–two of the main visual indicators of structural health after an earthquake. As the distribution of plastic strain is not likely to change for small changes in the loading, the number of analyses can be further reduced for large structures with little effect on the final result (i.e., the rendered PBGM) by taking advantage of the fact that components often repeat in a structure (e.g., across floors in a building). The next subsection describes the proposed methodology to identify physics-based damage hotspots using non-linear analysis. 

### 2.3. Damage Masks Generation

Damage masks are 2D binary images that indicate the presence of damage on component surfaces. Several damage parameters need to be determined before these masks can be generated using the conducted analysis. These parameters relate to the number, size, shape, and location of the damage. Each of the relevant parameters may be determined through, (i) physics-based response, or (ii) defined heuristics. Both these modes come with their own set of merits and demerits. Heuristic methods are the only viable option for many damage cases that are difficult to model (e.g., due to lack of suitable material models or load representations) or for which no empirical data is available. Methods stemming from empirical data are reliable because they are based directly on observations but identifying good heuristics is challenging. When realizable, physics-based damage masks provide a rigorous approach that links the visual representation to results of finite element analyses, leveraging efforts by researchers in developing state-of-the-art constitutive models. Incorporating the physics enables applications such as estimating structural response (e.g., interstory drift, damage state, etc.), failure mechanisms, and model updating. We first propose a general framework to determine damage parameters and then demonstrate generating masks for common damage types of cracks and spalling using the structural response. 

The damage parameters are obtained by Monte Carlo sampling from empirical or heuristic distributions. The first step is to determine the damage state (*DS*) of the component based on some structural response measure δ (e.g., interstory drift). The response measure used may be anything that is sensitive to visual damage. For example, for reinforced concrete buildings with masonry infill, a commonly used damage indicator, the interstory drift may be used as the response measure. The relationship between *DS* and δ is then modeled through a probability distribution (1). This distribution represents uncertainties in the geometry, method of construction, and material properties. The component damage state $DS_{o}$ is determined by sampling from the distribution given by

(1) $$DS_{o}\sim P_{\delta }\left(DS\right)$$

Various parameters $q_{i_{0}}$ (e.g., number of cracks, crack width, crack length, etc.) are then calculated by sampling from their corresponding distributions representing variation in damage observed given a particular damage state shown in Equation (2).

(2) $$q_{i_{0}}\sim P_{DS_{i}}\left(q_{i}\right)$$

While it may be possible to estimate the parameters $q_{i_{o}}$ directly from δ, this two-step approach allows for a more intuitive method facilitating the construction of the distributions $P_{\delta }$ and $P_{DS_{i}}$ based on empirical data. For parameters whose value will vary depending on the location in the member, parameters are further modified by a multiplicative factor derived from the structural response as shown in Equation (3).

(3) $$Q_{i}\left(X,Y\right)=q_{i_{0}}f_{i}\left(X,Y\right)$$

where f is a function of some structural response parameter (e.g., plastic strain) varying in the component. Examples for selecting each of $P_{\delta }$, $P_{DS_{i}}$, and $f_{i}$ for RC buildings with masonry infill are provided in Section 3. The next subsections discuss the generation of masks for cracks and spalls–two common types of defects once the damage parameters have been determined. 

**Figure 7:**
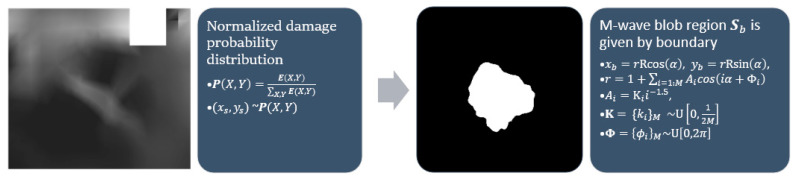
Stochastic blob generation.

Stochastic blobs are amorphous shapes generated to select subregions of generated masks. The plastic strain map E is normalized to take the form of a probability distribution P. A center point $\left(x_{s},\;y_{s}\right)$ is obtained by sampling from the distribution. An amorphous blob-shaped region $S_{b}$ is marked around the center point using a stochastic radius defined as the cumulative sum of a periodic function with random amplitude and phase. The blob generating function takes as input the number of waves along the circumference, w. The precise equations proposed can be found in Figure 7 where ~U[a,b] represents sampling from a uniform distribution between a and b.

**Figure 8:**
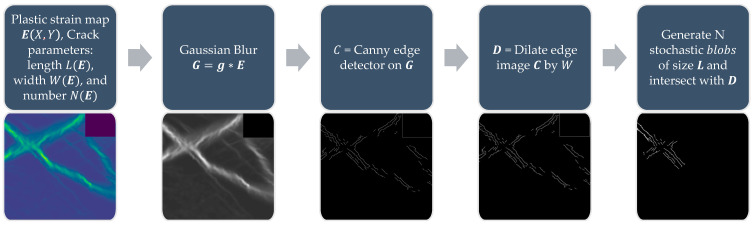
Crack mask generation process.

For each component, the set of crack parameters are $q_{o}=\left\{N,\;L,W\right\}$ where N is the number of cracks, L is the length of cracks in pixels, W is the crack widths in pixels. Once these parameters are determined, the following pipeline for the generation of crack masks from the plastic strain map provided in Figure 8 can be applied. A gaussian blur, with a kernel g is applied, followed by a Canny edge detector [39] to obtain an edge image. The edges are dilated by a factor of W. Finally, to add randomness to each component, a stochastic blob is generated and the intersection of the blob with the dilated edge image is included in the crack mask. This process is repeated N times. 

**Figure 9:**
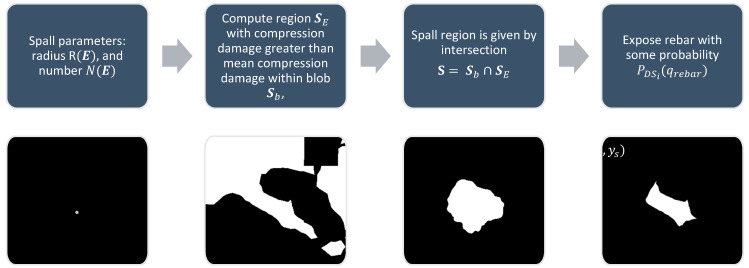
Pipeline for generation of spall masks from plastic strain.

Spalling is another common damage type for reinforced concrete and masonry structures affecting the integrity of components. The damage parameters to be determined here are $q_{o}=\left\{N_{s},\;R\right\}$ where $N_{s}$ is the number of spalled regions, and R is the nominal spall radius. To generate spall masks with these parameters, an area of pixels corresponding to the spall must first be defined. A stochastic blob $S_{b}$ is generated following the process outlined in Figure 7. In addition to the blob, another region $S_{E}$ is constructed corresponding to pixels with compression damage greater than the mean compression within the blob. The spall region S is then set as the intersection of $S_{E}$ and $S_{b}$. Rebar is made visible under spalled regions with some probability $P_{DS_{i}}\left(q_{rebar}\right)$. The process is illustrated in Figure 9.

### 2.4. Damage Textures

Damage textures are image textures of damaged components. Damage textures need to be generated so as to provide a realistic visual representation of the damaged structure. The following points are discussed to illustrate the process followed in generating damage textures: (i) Bidirectional scattering distribution functions, (ii) material textures, (iii) damage textures, and (iv) annotation textures.

Bidirectional scattering distribution functions: The visual appearance of an object is dependent on how light incident on its surfaces is scattered, transmitted, or reflected. In computer graphics, the behavior of light incident on a given material is represented by a bidirectional scattering distribution function or BSDF [40]. BSDFs can be measured through optical experiments with structured light sources. Based on experiments, researchers have proposed different methods to model BSDFs. A widely implemented model available in many 3D creator software known as the Principled BSDF was proposed by Burely et al. [41] and is a physically-based rendering (PBR) model but with artistically intuitive parameters. Apart from the base color, BSDFs have 10 parameters to describe each pixel including properties of roughness, metallic, specularity, anisotropy, etc. Depending on the type of material, several of these may not be applicable, for example, a concrete surface may have negligible metallic scattering properties. In addition to these values defining the scattering, the incorporation of surface normal directions at every point plays a significant role in accurate renderings. If the surface is modeled at a very small-scale incorporating undulations, then the values of the surface normal can be computed directly from the geometry. However, such detailed surface modeling is seldom feasible and an alternative way to retrieve the same effect is to use a predefined surface normal map. 

**Figure 10:**
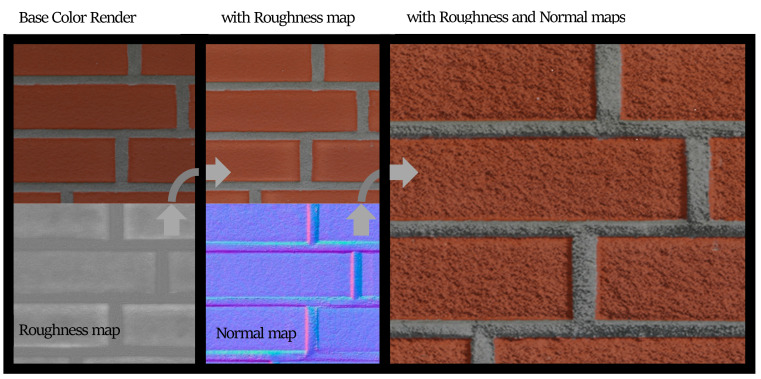
Illustration of PBR texture using base color, roughness and normal maps.

Material textures: PBR textures encompassing maps with BSDF parameters for the base color, roughness, metallicity, etc., and surface normals can be used to adequately represent materials for the purpose of structural inspection simulation. PBR textures for common construction materials incorporating BSDF parameters created through height field photogrammetry are available on websites like CC0textures [42]. A sample image texture of a brick wall rendered from [42] using Blender [43], an open-source 3D model creation software is shown in Figure 10. The example incorporates three maps: the base color, a roughness map, and the normal map. The roughness changes how the light is reflected, especially near the edges of the bricks and the normal map helps visualize the fine surface undulations and the protrusion of the bricks from the mortar plane. In addition to photogrammetry-based textures, textures can also be procedurally generated in material authoring programs like Substance [44] and provide the ability to create multiple textures with different random seeds. As noted in [29,38] randomization is a crucial means of enforcing generalization. We utilize both types of PBR maps (photogrammetric and procedural) in the construction of the PBGMs. When multiple layers of materials are present, (e.g., cement plaster over masonry, paint over concrete, etc.) maps are selected for each material layer, and the displayed layer is selected based on the presence of damage at any given pixel. 

Damage textures: The damage textures for the PBGM are obtained using the material textures as the base and modifying the region within the generated physics-based damage masks using opencv-Python [45]. The crack is textured by modifying the corresponding surface normal through a bump map. The depth is set as a heuristic function of the plastic strain similar to the width and length and the crack. The spall region is defined by applying a Musgrave [46] texture to create a bump map controlling the variation of depth within the spall region. For reinforced concrete components, rebar is exposed depending on the damage state of the material with some probability p. The rebars are modeled as cylinders with surface variation and a metallic map. 

Annotation textures: For deep learning methods, the ground truth synthetic data is rendered by using an emission BSDF. As opposed to the principled BSDF with 10 parameters, an emission BSDF has a single color parameter and acts as a light source. The emission shader is useful for rendering homogenous colors, which is what is required as ground truth for tasks like semantic segmentation. Depending on whether image data or annotation data is being rendered, the appropriate texture types are selected during the rendering process.

**Figure 11:**
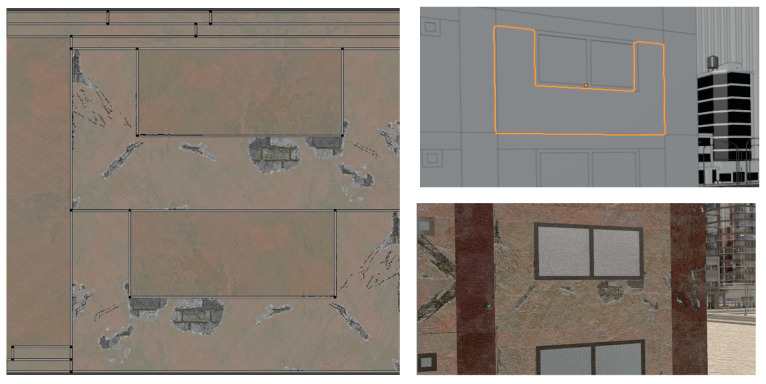
UV unwrapping and texture assignment to a wall.

The generated textures are applied to the components after UV unwrapping the components. For 3D models application of 2D textures requires a correspondence to be created such that 2D surfaces can map to corresponding locations on the 3D surface. This process of “unwrapping” the 3D model is termed UV unwrapping. UV unwrapping is conducted by selecting the edges that are to serve as seams to break up the 3D model. In most programs, once the seams are selected, the resulting 2D surfaces are then arranged to fit within a square surface. The obtained damage masks are also assembled in the same arrangement to create a direct correspondence to the UV map and thus to the 3D model. Other masks like the rebar mask are also arranged in the same way. Here, depending on the aspect ratio of the component, the arrangement can take on a handful of configurations that are hard coded along with the dimensions of the corresponding component so that the rest of the process can be automated. An example of a UV unwrapped image is provided in Figure 11.

### 2.5. Scene, Lights, Camera & Rendering

The steps discussed thus far describe the construction of a single PBGM. To obtain photo-realistic images, the background scene also needs to be populated. For post-earthquake building inspections, which include multiple buildings, roads, sidewalks, light poles, electric cables, trees, etc. Randomization of geometry and textures is important towards the ultimate goal of the generalizability of deep learning models trained in the synthetic environment. Thus procedural methods are adopted even in the scene assembly for the generation of these items. 

The final step is to render the images. There are two modes of rendering commonly available in 3D creator software, namely path tracing and rasterization. Path tracing involves simulating the path of light in the scene and is more computationally expensive than rasterization but is preferred as it produces more photorealistic representations. To render images, a light source and the camera locations and orientations are to be set in the synthetic environment. To simulate realistic outdoor lighting, HDRI maps are used to light the scene [47]. 

## 3. Implementation of 3D Synthetic Environment with RC Buildings

The proposed framework was implemented using multiple software applications. 3D model construction was conducted in Blender 2.9 [43], the finite element analysis was conducted using ABAQUS [48] and OpenSeesPy [49], material authoring using Substance [44], and image processing in Python using OpenCV [45]. A summary of the applications used are provided in Table 1. This section discusses parameters used for the construction of the synthetic environment. While considerable care was taken in the selection of parameters, detailed studies on parameter selection are out of the scope of this manuscript and will be the subject of future research. 

### 3.1. Graphics Mesh

**Figure 12:**
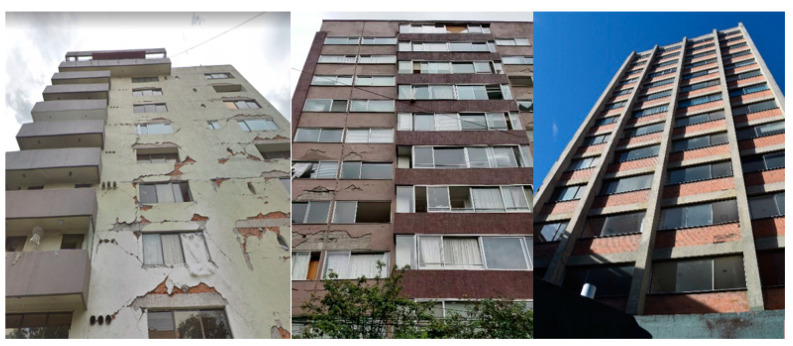
Three reference buildings damaged that suffered damage during the Mexico City earthquake in 2017.

3D models were created for 12 different fictional reinforced concrete buildings with masonry infill walls. The main reason for creating multiple buildings is to be able to generate diverse data that can be used for further experiments. The layout for these buildings was loosely 3 buildings (shown in Figure 12) that were affected in the Mexico City earthquake in 2017, with some simplifications made for parametric modeling. The buildings were parameterized and different realizations for each of the buildings were constructed with varying dimensions. Photographs of the buildings were obtained from three different sources: datacenterhub [50], Google Street View [51], and direct photography by the authors. 

The dimensions and layout of the building were parameterized to include dimensions and locations of columns, beams, walls, windows, and balconies. The building properties were stored in a single class object that were used both for finite element model creation and 3D model generation. 

### 3.2. Non-Linear Finite Element Analysis 

As mentioned in Section 2, both the global and local responses of the structure are required for the generation of the PBGM. The global analysis of the buildings was conducted using OpenSeesPy. The creation of the mesh was automated based on the building layout parameters developed in the previous section. The structure was modeled using the confined concrete model in OpenSeesPy with the parameters in Table 2. 

Reinforced concrete sections were created for the members using the patch, section, and layer commands in OpenSeesPy. The amount of rebar was set based on ACI 318-14 [52] assuming a c/d ratio of 0.3 in Equations (4) and (5), where $A_{r}$ is the reinforcement area, b,d are the section dimensions of the concrete member, ρ is the rebar ratio, β=0.85, ${f_{c}}^{\prime }$ and $f_{y}$ are the yield strengths of concrete and steel. A concrete cover of 40 mm was set ACI minimum of 1.5 in for beams and columns.

(4) $$A_{r}=\rho bd$$

(5) $$\rho =0.85\beta \frac{c}{d}\frac{f_{c}^{\prime }}{f_{y}}$$

The shear reinforcement was assumed to be at a spacing of a maximum of (100, d/3). The shear strength from reinforcement is assumed to be $3\sqrt{f_{c}^{\prime }}bd$ and the corresponding rebar area as per ACI 318 are given by

(6) $$A_{v}=\frac{3\sqrt{f_{c}^{\prime }}bs}{f_{y}}$$

**Figure 13:**
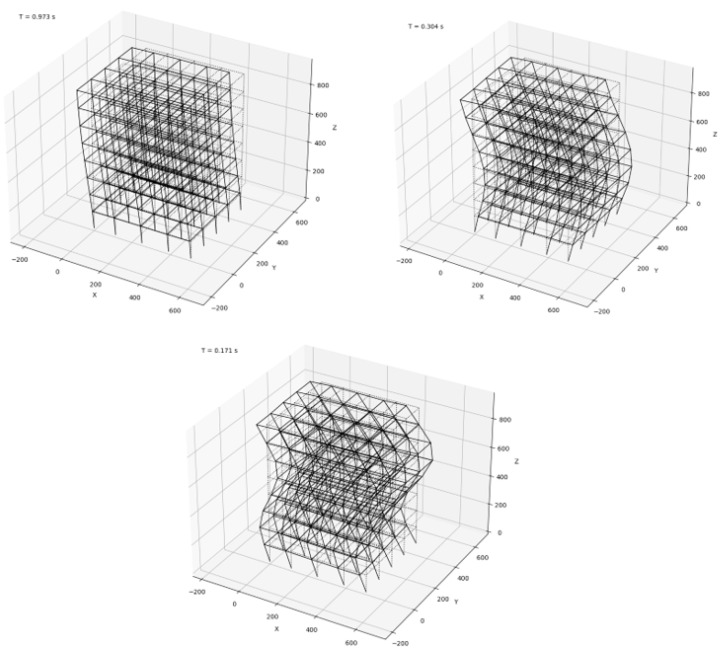
First three horizontal modes of a simulated structure.

The first three global mode shapes of a parametrically generated building are shown in Figure 13.

**Figure 14:**
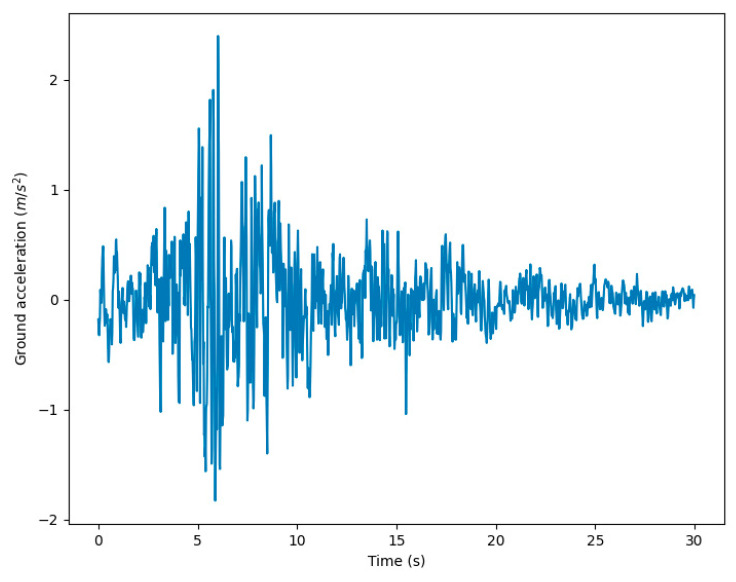
Ground motion input to global model with PGA g/4.

**Figure 15:**
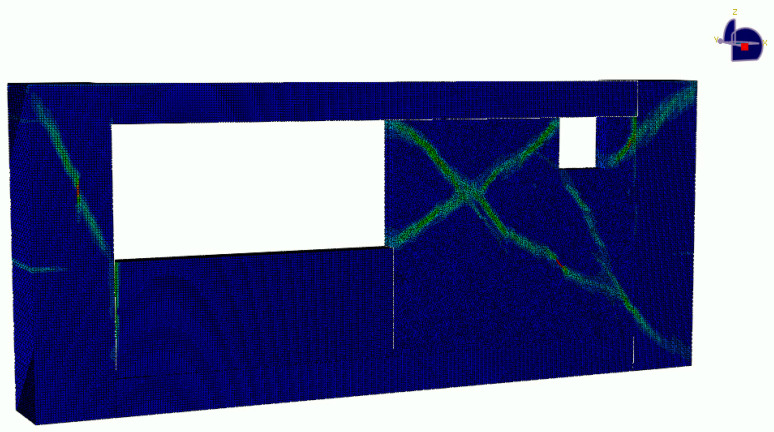
Illustrative response of component model in Abaqus including walls with window openings, columns and beams.

**Figure 16:**
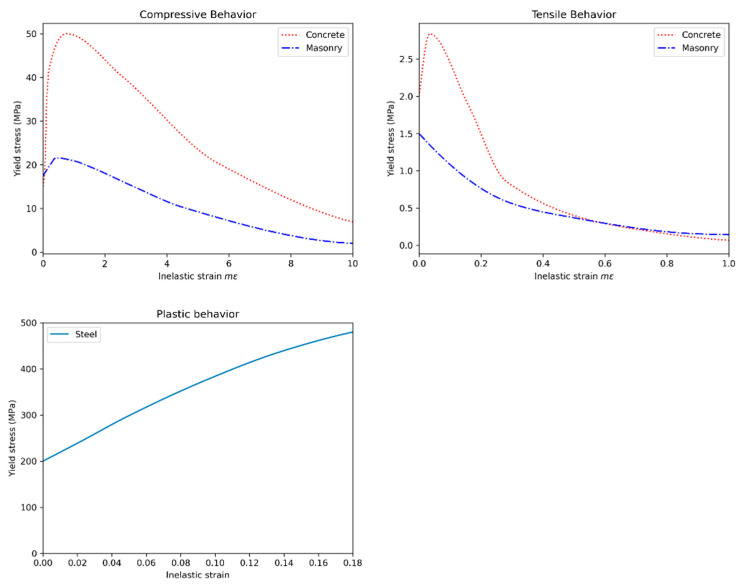
Plastic stress strain curves for concrete, masonry and steel used in the PBGMs.

Each building was subject to the Tabas earthquake with varying intensity from g/4 to g/6 from both x and y directions. An example of ground motion is shown in Figure 14. A full analysis was conducted for the local response of the components using Abaqus. A Python script was developed to automate the process of creation of the components of the structure. The components models included the masonry wall and confining columns and beams as shown in Figure 15 all modeled with solid elements. The model also included rebar which was modeled with beam elements with a circular cross-section. The nodal displacements at the corners of components were used as inputs for the detailed local component models. For the concrete and masonry members, the concrete damaged plasticity (CDP) model proposed by [53] was used. The material parameters used for the concrete material were based on values reported in Jankowiak et al. [54] and for the masonry material based on the values reported in Bolhassani et al. [55]. The masonry yield stress for tensile behavior was factored down so that the tensile strength of the masonry was less than that of the concrete. The steel was modeled as a plastic material with a yield stress of 200 MPa. The stress-strain curves used are shown in Figure 16.

The rebars are embedded within the concrete members using the embedment interaction option in ABAQUS. The walls are tied to their immediate confining members using the tie constraint in Abaqus. A multi-point constraint is applied to tie the top and bottom surfaces of the beams and columns together. The bottom surface is fixed, and the top surface is subject the interstory drift. The amplitudes are chosen to represent 4 different damage states derived based on values reported by [56]. 

An Abaqus explicit analysis was run for each unique component and the plastic strains at each of the amplitude levels are stored as an image for input to the texturing process discussed in the next subsection.

### 3.3. Damage Mask Generation

**Figure 17:**
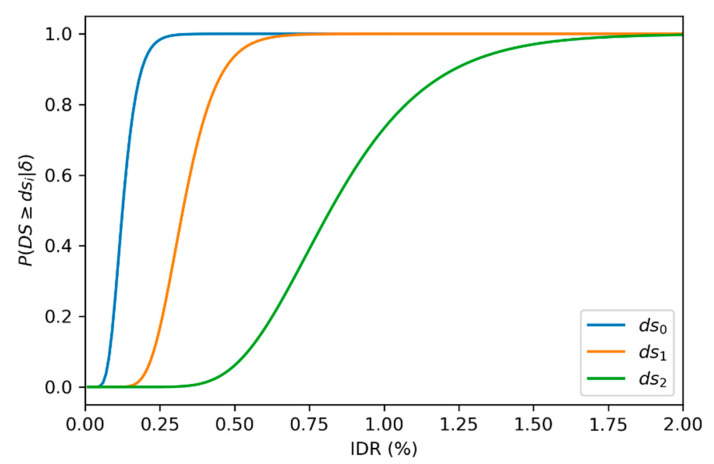
Damage states fragility curves.

As mentioned in Section 2, the first step in identifying the damage parameters is to determine the damage state of the component. The probability distribution for different damage states given the interstory drift is taken from Chozzi et al. [56], where data from over 150 tests on masonry walls subject to in-plane loading were analyzed. A log normal distribution is used to model the conditional probability of exceeding a given damage state as shown in Equation (7). $\mu_{ln}\left(\delta \right)$ and β represent the central tendency and the dispersion parameters of the cumulative standard normal distribution Φ. The values used for the different damage states are presented in Table 3, and the corresponding curves are plotted in Figure 17.

(7) $$P\left(DS\geq ds_{i}|IDR=\delta \right)=1-\Phi \left(\frac{\ln\left(\delta \right)-\mu_{ln}\left(\delta \right)}{\beta }\right)$$

**Figure 18:**
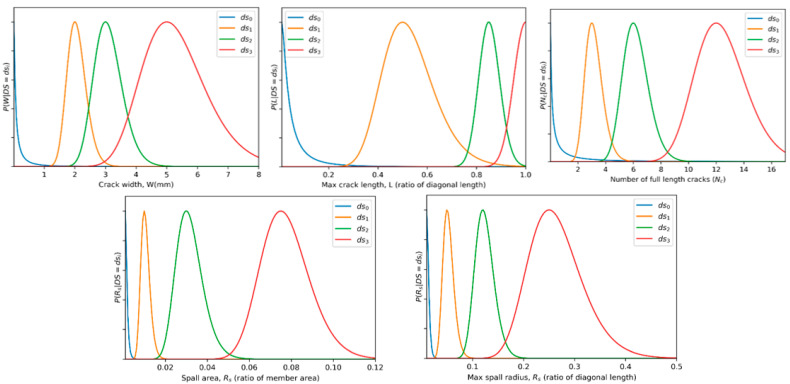
Visualization of damage parameter distributions.

Once the damage is determined for the components, the various damage parameters were computed by sampling from their corresponding lognormal distributions. The statistics of the distributions used are provided in Table 4 and the corresponding distributions are plotted in Figure 18. The values for the crack width are based on descriptions of damage states given in Chozzi et al. [56]. The crack length, height, and number of cracks for different damage states are approximated based on descriptions given in FEMA 306 [57] based on the component damage classification guides for concrete frames with masonry infill. The spall radius ratio $R_{s}$ and area $A_{s}$ has been generalized for both walls and columns based on examples provided in [58]. In the presence of more rigorous experimental data, corresponding distributions may be replaced to better represent the damaged structure.

### 3.4. Damage Texture Generation

PBR textures are used for all the construction materials. The textures for the paint, walls, beams, and columns were all generated parametrically using Adobe Substance Designer [44]. The visual features parameterized include color properties, amount of dirt, types of dirt, and size and orientation of bricks. For each generated building structure, parameters including the paint color, concrete color, brick size, and brick color are first selected. Then for each component, the parameters are perturbed to provide variability for the components. 

### 3.5. Scene, Lights, Camera & Rendering

The assembly and construction of the PBGM and synthetic environment are automated using Python scripts. In each scene, one PBGM building is created. Then, the sidewalks, trees, roads, and other buildings are added to complete the scene using the SceneCity Blender plugin. The scene background and lighting was set using HDRI maps downloaded from [42]. An emission shared was used for the annotations, and the images were rendered using the cycles renderer. 

## 4. Experiments and Results

The developed procedure for PBGMs is used to generate synthetic images that can be used for automated visual inspection studies. Three applications and examples are illustrated, (i) QuakeCity Dataset: Large-scale synthetic dataset of earthquake-damaged buildings, (ii) Augmenting real data with synthetic data, and (iii) Comparing post-disaster UAV data acquisition with ground camera data acquisition.

### 4.1. QuakeCity Dataset: Large-Scale Synthetic Dataset of Earthquake Damaged Buildings

Images are rendered from multiple simulated UAV surveys of 11 damaged buildings in a city environment to create a new dataset called QuakeCity. Each survey replicates a field scenario where a UAV circles the building at different altitudes to cover the entire height, width, and length of the building. Each image captured by the simulated UAV is associated with six different sets of annotations, including three damage masks (cracks, spalling, exposed rebar), components, component damage states, and a depth map. In total, 4688 images and six annotations per image of size 1920 × 1080 are included in the dataset, with 3684 for training, and 1004 for testing. 

**Figure 19:**
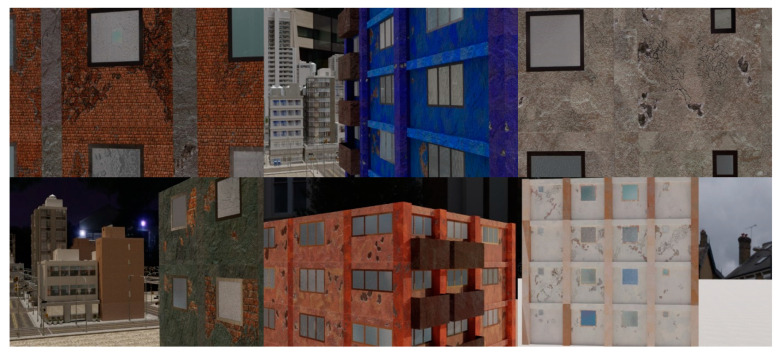
Example images from the QuakeCity Dataset.

**Figure 20:**
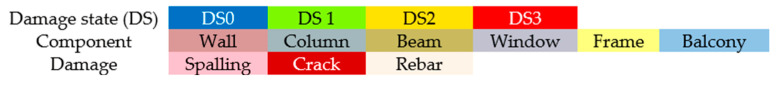
Annotation color key.

**Figure 21:**
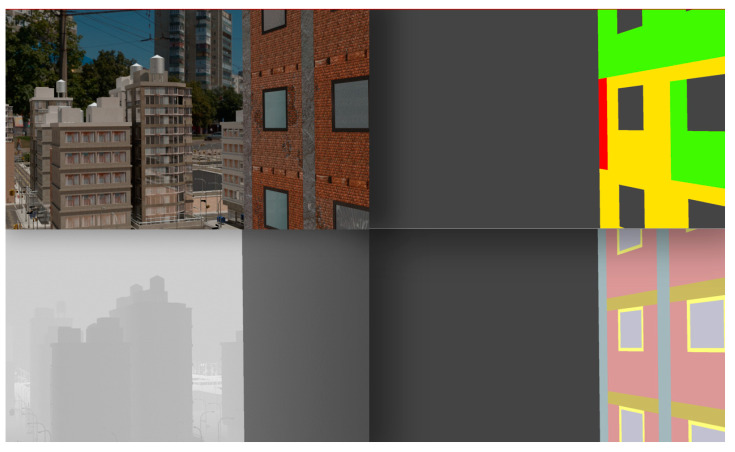
Example annotations (clockwise from top left) Image, Component Damage State, Components, Depth.

**Figure 22:**
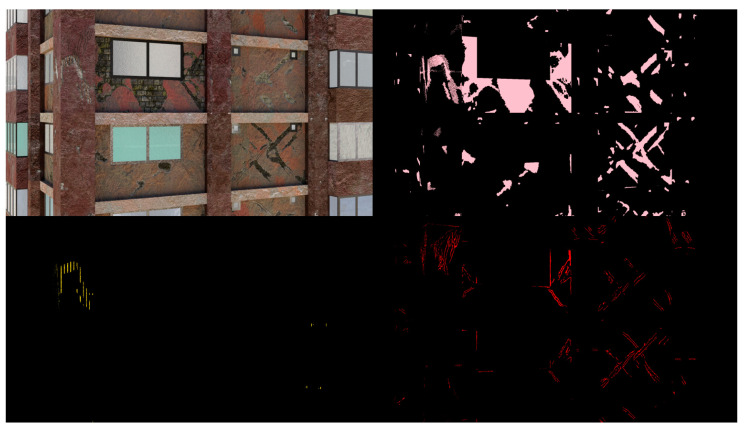
Example annotations (clockwise from top left) Image, Spalling, Cracks, Exposed Rebar.

Example images of the generated dataset are shown in Figure 19. The images demonstrate the diversity of damaged buildings in the dataset in terms of layout, color, damage level. Images in the scenes are taken from different viewpoints and with different lighting conditions. Each image in the dataset has six annotations and the color key for annotations are provided in Figure 20. Figure 21 shows three example annotations including component damage state, depth map, and component annotations. Figure 22 shows another image generated with spalling, cracks, and rebar annotations for each pixel. 

### 4.2. Augmenting Real Data with Synthetic Data

To reliably train an autonomous visual inspection system, a large amount of training data with damaged structures would be required. Frequently, however, the amount of such training data available is limited. Additionally, careful annotation of available images is also a challenge. In this experiment, we are interested in studying whether the incorporation of synthetic data in cases with limited availability of real data with annotations can help boost the accuracy of networks on unseen real data. 

#### 4.2.1. Real Image Dataset

A dataset for semantic segmentation of real earthquake-damaged buildings was developed for the purpose of this study. The images were acquired by the authors after the 2017 Mexico City Earthquake using a DJI Phantom 3, and a Nikon D3300. The images were annotated for the presence of spalling using InstaDam [59]. In total, 150 images of resolution 1920 × 1080 were annotated as part of the dataset.

#### 4.2.2. Network Architecture

**Figure 23:**
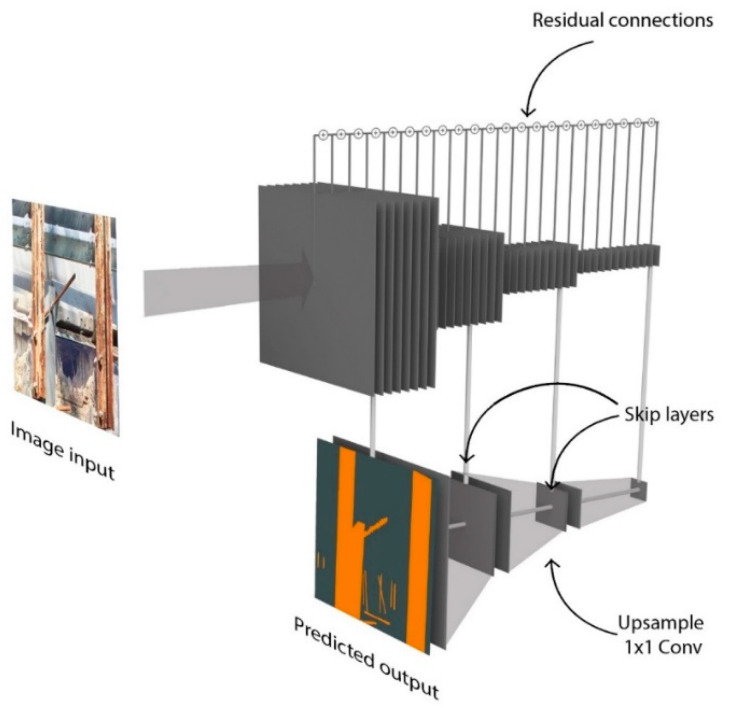
Schematic illustration of feature layers in the proposed FCN.

A deep network is constructed for semantic segmentation using a ResNet [60] architecture with 45 layers. The details of the encoder part of the architecture are provided in Figure 23. Residual connections involve the summation of the output of prior layers to enforce learning of new information in subsequent layers. These residual connections are used between alternate layers (e.g., Conv0 to Conv2, Conv2 to Conv4, etc.). A rectified linear unit is used as the non-linearity for all layers of the network. The details of the decoder part of the architecture are provided in Model training. The skip connections with 1 × 1 convolutions described in the previous subsection are taken after the Conv8, Conv20, and Conv32 layers. The network parameters were trained by minimizing the cross-entropy loss function between the predicted softmax probabilities and the corresponding one-hot labels with an L2-regularization weight decay [53]. The incorporation of the weight decay term gives preference to smaller weights and helps tackle overfitting. Batch normalization was applied to address the covariate shift that occurs during training [24], where each feature dimension is shifted by a weighted mean and standard deviation that was learned during training. The percentage of pixels in each of the classes varies significantly. For example, some classes such as cracks have much fewer pixels than spalling or corrosion due to the nature of the damage. To balance the frequencies of different classes in the data set and prioritize all classes equally, median class balancing [26] was applied by reweighting each class in the cross-entropy loss. Data augmentation by resizing and cropping was incorporated in order to increase the efficacy and efficiency of training and prevent issues such as overfitting. The training was conducted using the Adam optimizer [54] implemented in Pytorch [61].

#### 4.2.3. Model Training

Eight different models were trained to evaluate the potential role of synthetic data in enhancing the overall performance of the models on real data. The eight models included four pairs of training schemes listed in Table 5, where each scheme had one model trained purely on real data and another trained on real plus synthetic data. In each pair, the train/test split of real data was varied, starting from 0.2 train + 0.8 test, to 0.8 train + 0.2 test, in increments of 0.2. The same amount of synthetic training data was used in all four schemas, and this included the training images from the QuakeCity dataset (i.e., 3684 images). 

**Figure 24:**
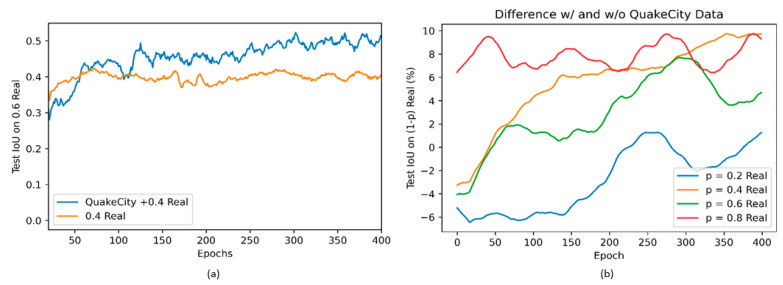
(**a**) Comparison of test set accuracy on 60% of real data while training on 40% of the real images with and without QuakeCity data (**b**) Difference between test accuracy with and without QuakeCity data for varying fractions of real training data.

The results from the different models trained are shown in Figure 24a,b. Figure 24a shows the comparison of test Intersection-over-Union (IoU) [62] on 60% of real data while training on 40% of the real images with and without QuakeCity data. While the initial accuracy with only real data is higher than with QuakeCity, after about 75 epochs, it was noticed that there was a significant increase in the performance of the model trained with QuakeCity data. The performance of the model clearly highlights the benefits of using synthetic data to improve the performance of deep learning models on unseen real data. 

The addition of synthetic data was also shown to improve the performance of the deep neural network even for varying splits of training and testing data. Figure 24b shows the difference between the two values plotted in Figure 24a, for all four models trained. The performance of all models trained with the QuakeCity dataset is better than the model without the QuakeCity data after 400 epochs. The improvement in IoU is seen to be as much as 10%. Table 6 shows examples of images where the 0.4 Real model with QuakeCity data performs better than the model without. The quality of the predictions is clearly improved, and the border of the predictions can be seen to be more accurate. 

### 4.3. Comparing Damage State Estimation Using UAV and Ground-Based Images

While implementing autonomous visual inspection systems after disasters, a trained model using a dataset conducted prior to the disaster would be used to process new data acquired after the disaster. The quality of the predictions on new data may however vary widely depending on the image acquisition distance. For example, it may not always be possible to have consistent data acquisition modes or distances for various structures of interest. This is especially so in crowded cities where many obstacles are present. To better study the robustness of the trained models, practitioners may want to evaluate the model’s performance for different camera distances to see where data gaps are present in the model, or to inform their field acquisition strategies. In such a scenario, using a PBGM would prove very useful, as images could be acquired with different camera paths, and the accuracy of predictions of a fixed trained model can be studied. 

In this experiment, we train two different ResNet 45 models to predict component damage states. One model is using only the QuakeCity training dataset and is tested on images from another building. Two test sets are prepared, one simulating a UAV camera for data acquisition (UAV-B12), and another simulating a person on the ground collecting images of the structure by pointing the camera forward and upward (Ground-B12). Together, the datasets are referred to as B12. Another model is trained with the QuakeCity training dataset plus 25% of the images from B12 (QuakeCity + 0.25 B12) and evaluated on 75% of the B12 data (0.75 B12). The results of performance on the ground data are reported separately for the UAV and Ground parts of B12. 

Table 7 shows the test IoU for different damage states for the various models trained. The model trained on the QuakeCity dataset only, which is limited to UAV views performs poorly on Ground B12 images. As a comparison, the performance of the model on 75% of UAV B12 is also shown. With the addition of 25% of B12 to the training dataset, the model performs much better on the remaining 75% of the data and is much closer to the performance on 75% of the UAV B12 set. While the results are along expected lines, the study nonetheless highlights the benefits of using a PBGM for tasks where the value and type of additional information to be incorporated into the network needs to be quantified. Given that there will be some cost associated with incorporating new data into the training dataset, a performance-based approach for data inclusion can be set-up using a PBGM as a reference. Table 8 and Table 9 show examples of predictions for the Ground and UAV B12 test datasets, respectively. 

## 5. Discussion 

### 5.1. PBGM Parameters

An important aspect of generating effective PBGMs is the selection of various parameters in the five different steps of the proposed framework. The choice of these parameters will most certainly have an influence on the realism of the resulting simulations. In the absence of any data about the structure’s properties, several arbitrary assumptions were made in relation to the structure geometry, material properties, paint color, ground shaking intensity, etc., based on published literature. However, for autonomous inspection studies, the key requirement is the ability to generate large amounts of diverse data, and thus such assumptions, while not perfectly realistic, were reasonable for this research. 

### 5.2. Computational Cost

The proposed framework for data generation has several components that contribute to the relatively high computational cost of generating the data. Running non-linear time history analysis for each component of the structure is very computationally expensive. Leveraging the regularity of the plan in the buildings, and the fact that the same material model will produce similar responses at different floors for identically shaped components, the analysis was run for just one story with different intensities and re-purposed for use with other stories. This simplification greatly reduced the overall computational time. The 3D synthetic environment for one damaged building can be created in about 8 h of total time on a PC with 2 Nvidia RTX 2080 Ti, Intel i7-8700K, and 16 GB of RAM. The rendering of each image then takes about 1.5 min, and each annotation takes about 0.05 min using Blender Cycles. The distribution of time taken for each component of the framework is provided in Table 10.

### 5.3. Autonomous Inspection Experiments

The experiments conducted demonstrate the efficacy of the proposed framework as a testbed for end-to-end validation of autonomous inspections. 

The first experiment involved implementing the proposed framework to generate the QuakeCity dataset. The quantity and diversity of data generated in the QuakeCity dataset underscore the benefits of using 3D synthetic environments to generate data to study algorithms for autonomous inspections. While such studies were out of the scope of this manuscript, the dataset has been released as part of the International Competition on Structural Health Monitoring; over 150 teams of researchers are participating to study the performance of different algorithms with the dataset. 

The second experiment was conducted to study the utility of synthetic data generated from the proposed framework to directly augment deep networks trained for inference on real data for autonomous inspections. The results demonstrated that the use of synthetic data allowed the deep networks to learn better features that resulted in better performance on real data. The transferability of features learned on synthetic data to real data makes the use of the synthetic environment even more attractive. 

The third experiment illustrates another use case of the proposed framework to study the ability of already trained networks to perform on new scenarios. In the experiment, a deep network trained on UAV acquired data for physics-based damage state estimation is applied to data collected from the ground. The poor results, in this case, indicate that additional data would be required from a ground viewpoint to have an effective network. The addition of about 25% of the data from a single survey was found to increase significantly the performance of the network. Given the cost associated with acquiring data in the real world, such studies are crucial in efficiently developing inspection systems for use in field applications. 

## 6. Conclusions

This paper proposed a framework for generating physics-based graphics models (PBGMs) as part of a 3D synthetic environment that can support the development of automated inspection strategies for civil infrastructure. The proposed framework involved combining the response of a non-linear finite element model to inform the realistic visual rendering of different damage types. The framework was implemented for eleven reinforced concrete building structures subject to earthquake excitation and the damage types rendered included cracks, spalling, and exposed rebar. Three applications were demonstrated for the proposed framework. First, images were rendered from the damaged structures, pixel-level ground truth was generated for the various damage types, for components, component damage states, and depths. The QuakeCity dataset will serve as a benchmark dataset to study the use of deep learning algorithms in automated post-earthquake inspections of building structures. Second, the efficacy of the proposed framework in generating synthetic data to augment real data was demonstrated. It was shown that the performance of models trained with synthetic data and real data performed up to 10 IoU points better than models trained with only real data. Finally, a third experiment was conducted comparing the performance of trained models on the ground and UAV-based data. The experiment demonstrated the utility of the proposed framework for studying and quantifying the value of additional information for models trained for visual inspections. The results demonstrate the immense potential of using PBGMs as an end-to-end tool for the development and study of visual inspection systems. 

## Figures and Tables

**Table 1:** Summary of applications used.

Modeling Task	Application	Scripting
3D modeling and scene assembly	Blender 2.9	Python 3.x
Finite element (FE) modeling (local)	Abaqus	Python 2.7
FE modeling (global)	OpenSeesPy	Python 3.x
FE post-processing	OpenCV and misc. Python packages	Python 3.x
Texture generation	Substance Designer	Python 3.x
Image rendering	Blender Cycles	Python 3.x
Deep learning	PyTorch	Python 3.x

**Table 2:** OpenSeesPy model concrete material properties.

Material	${f_{c}}^{\prime }\;\mathrm{MPa}\;(\mathrm{ksi})$	${f_{cu}}^{\prime }\;\mathrm{MPa}\;(\mathrm{ksi})$	${\epsilon_{sc0}}^{\prime }$	${\epsilon_{sU}}^{\prime }$
Concrete01	41.36 (−6)	−34.4 (−5)	−0.004	−0.015

**Table 3:** Interstory drift ratio for different damage states.

Damage Description	Low	Moderate	Severe
Interstory drift ratio (IDR) %	0.125	0.25	0.82

**Table 4:** Statistics for damage parameters.

Damage Parameter	μ				β			
Damage State	$ds_{0}$	$ds_{1}$	$ds_{2}$	$ds_{3}$	$ds_{0}$	$ds_{1}$	$ds_{2}$	$ds_{3}$
Crack width W (mm)	0.01	2	3	5	1.5	0.15	0.15	0.2
Crack length ratio L	0.01	0.5	0.85	1	1.1	0.2	0.05	0.05
Number of cracks $N_{c}$	0.01	3	6	12	2	0.2	0.15	0.15
Spall radius ratio $R_{s}$	0.001	0.01	0.03	0.07	0.5	0.2	0.2	0.15
Spall area ratio $A_{s}$	0.01	0.05	0.12	0.25	0.3	0.2	0.15	0.2

**Table 5:** Training schemes evaluated.

Training Scheme	Number of Real Images	Number of Synthetic Images	Test Set (Real Images)
0.2 Real	30	0	120
0.2 Real + QuakeCity	30	3684	120
0.4 Real	60	0	90
0.4 Real + QuakeCity	60	3684	90
0.6 Real	90	0	60
0.6 Real + QuakeCity	90	3684	60
0.8 Real	120	0	30
0.8 Real + QuakeCity	120	3684	30

**Table 6:** Qualitative comparison of results with and without QuakeCity training data.

Real Image	Ground truth	Real 0.4	QuakeCity + Real 0.4
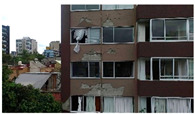	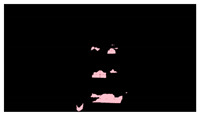	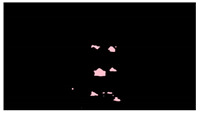	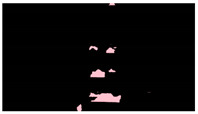
IoU	N/A	0.40838659	0.84830129
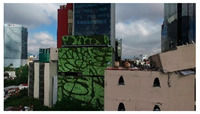	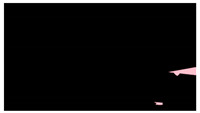	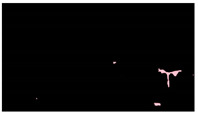	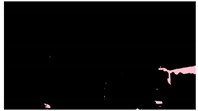
IoU	N/A	0.23776706	0.60649584
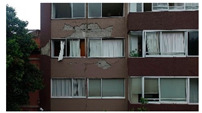	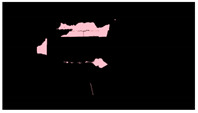	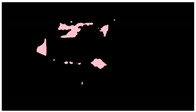	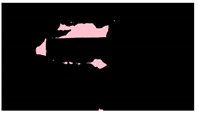
IoU	N/A	0.56585139	0.89356358
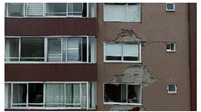	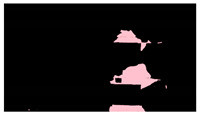	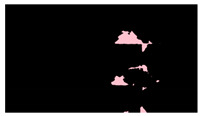	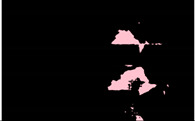
IoU	N/A	0.3649658	0.65546518
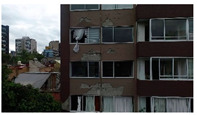	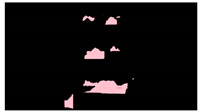	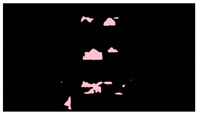	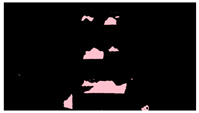
IoU	N/A	0.60729252	0.89446025
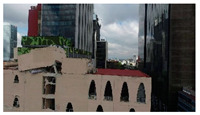	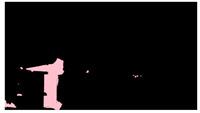	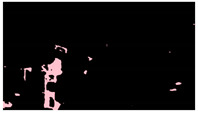	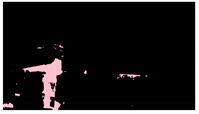
IoU	N/A	0.54610351	0.76673772

**Table 7:** Comparing damage state estimation using UAV and ground-based images.

Train	Test	ds1	ds2	ds3
QuakeCity	Ground B12	0.08	0.49	0.10
QuakeCity + 0.25 B12	0.75 UAV B12	0.40	0.72	0.79
QuakeCity + 0.25 B12	0.75 Ground B12	0.40	0.65	0.71

**Table 8:** Results for Ground B12 images.

Image from Ground B12	Ground Truth	Inference
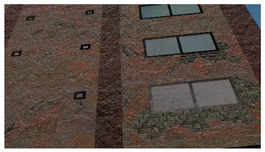	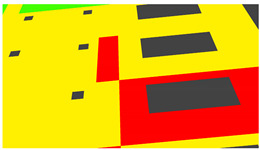	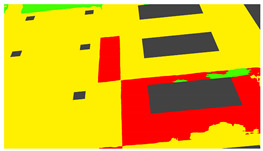
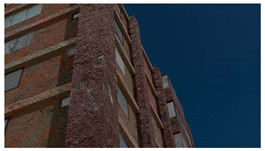	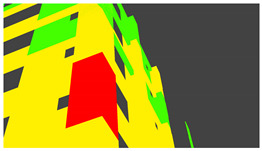	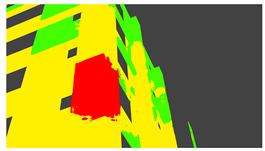
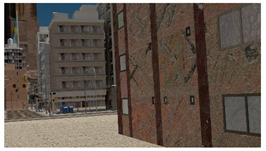	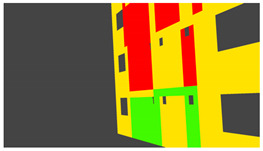	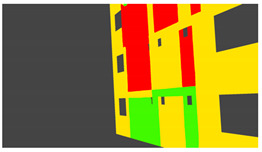

**Table 9:** Results for UAV B12 images.

Image from UAV B12	Ground Truth	Inference
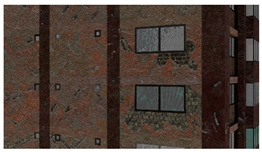	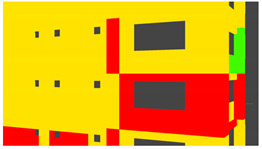	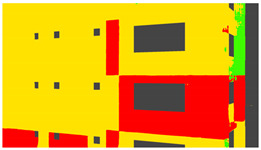
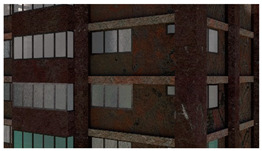	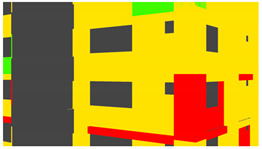	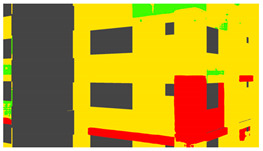
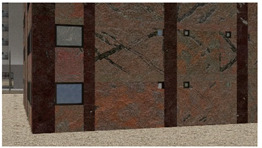	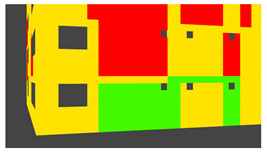	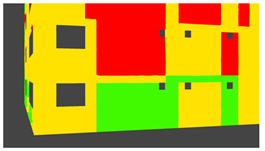

**Table 10:** Computational cost for PBGM generation.

	Time Taken (minutes)
Graphics Mesh (per building)	0.5
Global FE analysis (per building)	15
Component-level (single story)	360
Damage texture generation (per building)	25
Scene assembly (per environment)	4
Image rendering (per image, 1920 × 1080)	1.5
Annotation rendering (per annotation)	0.05

## Data Availability

The dataset generated, termed QuakeCity, was released as part of the International Competition on Structural Health Monitoring 2021.
